# Supplementary material for: A Language Vision Model Approach for Automated Tumor Contouring in Radiation Oncology
Source: Bioengineering (Basel). 2025 Jul 31;12(8):835. doi: 10.3390/bioengineering12080835 (PMC12383427; doi:10.3390/bioengineering12080835)
Supplement: Supplementary file 1 [file bioengineering-12-00835-s001.zip › bioengineering-3774372-supplementary.pdf]

Supplementary Materials:

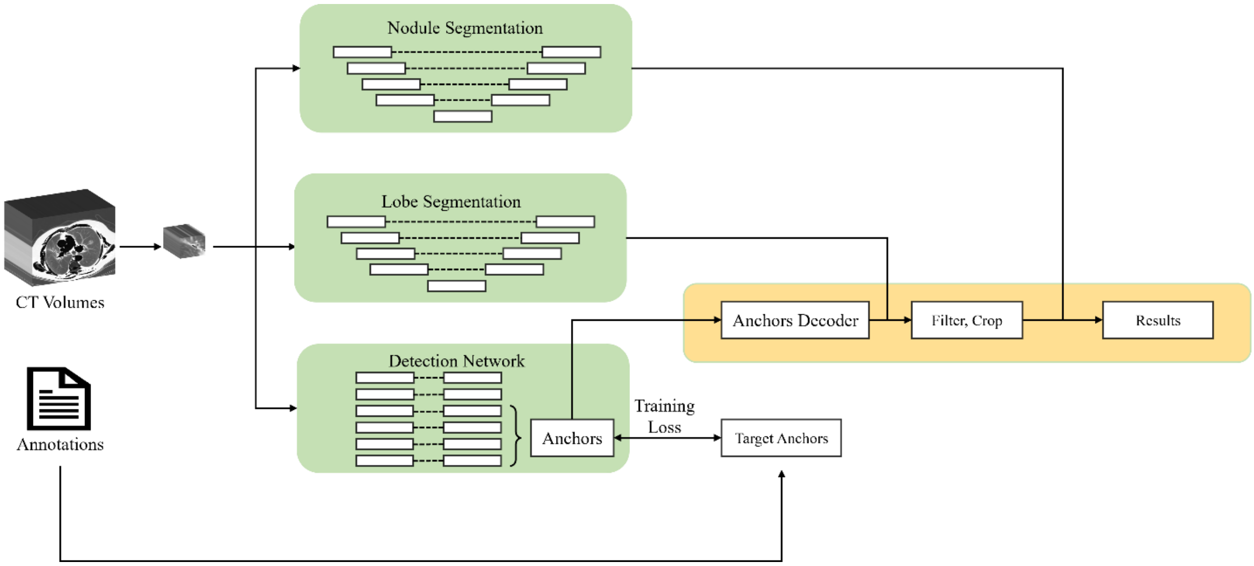

Figure S1: Structure of Retina-Unet3D.

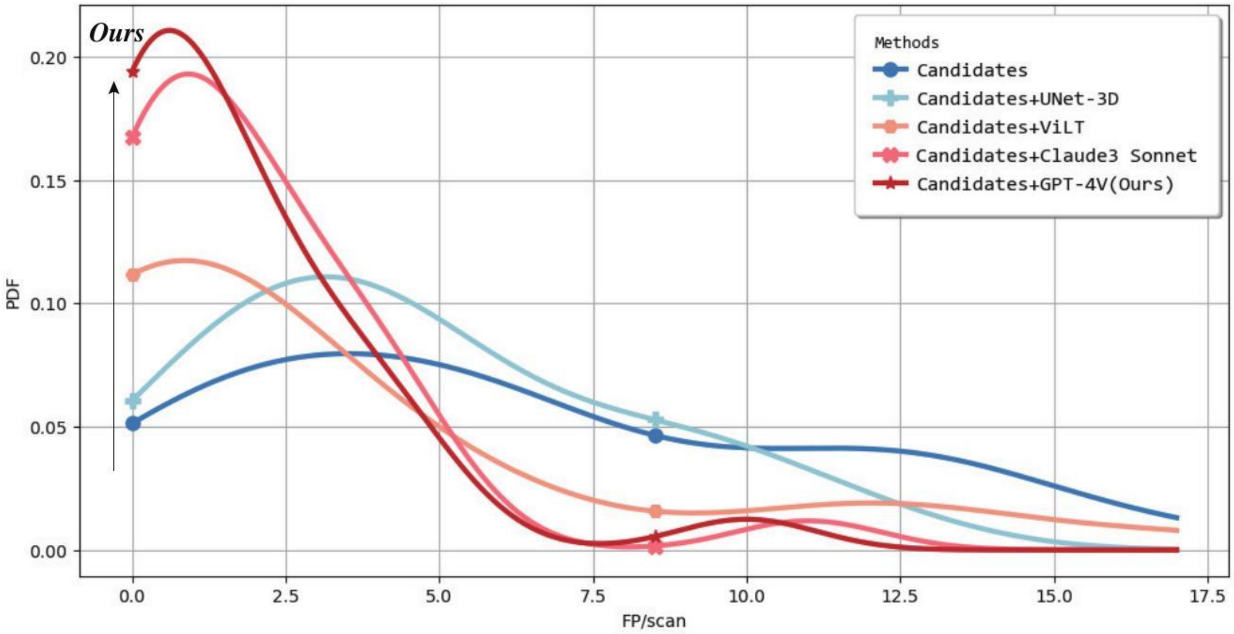

Figure S2: PDF curves comparing different methodologies for false positive reduction.
